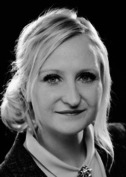# Photo‐biocatalytic Cascades: Combining Chemical and Enzymatic Transformations Fueled by Light

**DOI:** 10.1002/cbic.202000587

**Published:** 2020-11-06

**Authors:** Fatma Feyza Özgen, Michael E. Runda, Sandy Schmidt

**Affiliations:** ^1^ Groningen Research Institute of Pharmacy Department of Chemical and Pharmaceutical Biology Antonius Deusinglaan 1 9713 AV Groningen (The Netherlands

**Keywords:** biocatalysis, chemoenzymatic cascades, cofactor recycling, enzymes, photo-biocatalysis

## Abstract

In the field of green chemistry, light – an attractive natural agent – has received particular attention for driving biocatalytic reactions. Moreover, the implementation of light to drive (chemo)enzymatic cascade reactions opens up a golden window of opportunities. However, there are limitations to many current examples, mostly associated with incompatibility between the enzyme and the photocatalyst. Additionally, the formation of reactive radicals upon illumination and the loss of catalytic activities in the presence of required additives are common observations. As outlined in this review, the main question is how to overcome current challenges to the exploitation of light to drive (chemo)enzymatic transformations. First, we highlight general concepts in photo‐biocatalysis, then give various examples of photo‐chemoenzymatic (PCE) cascades, further summarize current synthetic examples of PCE cascades and discuss strategies to address the limitations.

## Graphical abstract

**Light'em up**! The combination of photo‐chemocatalytic and biocatalytic transformations represents an elegant strategy to extend the photo‐biocatalytic toolbox for the synthesis of various pharmaceuticals and fine chemicals. This review presents recent achievements in the field and highlights promising strategies to overcome incompatibility issues.

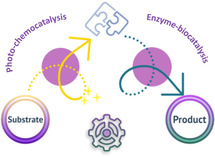

## 1 Introduction

By combining several catalytic steps into a precisely arranged sequence of chemical transformations in a single reaction vessel, the synthesis of complex molecules from much simpler precursors becomes feasible. Such so‐called multicatalytic cascade reactions not only exhibit an enormous potential to minimize downstream and purification steps but can also lead to a drastic reduction of the produced waste. Moreover, if all catalysts and reagents are present from the beginning of the reaction (this type of reaction is referred to as concurrent cascade or tandem reaction), cascades often allow minimized production times. Furthermore, cascade reactions not only facilitate steps that generate unstable or toxic intermediates but allow to perform thermodynamically challenging reactions that would be difficult to realize if performed as a single reaction.[^1^, ^2^] Thus, it is not surprising that the development of such one‐pot (cascade) reactions is a growing research field.[^3^, ^4^, ^5^, ^6^, ^7^, ^8^] Not only in the area of biocatalysis,[^3^, ^9^, ^10^, ^11^] but also within the fields of homogeneous‐,[^12^, ^13^, ^14^] heterogeneous,[^5^, ^6^, ^15^, ^16^] organo‐^[17]^ and photocatalysis^[18]^ cascade strategies have been successfully developed. Despite the beauty of many (chemo)enzymatic cascade reactions, the combination of catalysts from different fields can be challenging due to compatibility reasons.[^7^, ^19^] For instance, the majority of enzyme‐catalyzed reactions require aqueous conditions, whereas many reactions employing transition metal catalysts or organocatalysts require organic solvents. Overcoming these compatibility problems opens up the possibility to obtain products resulting from cascades that combine different chemistries which would not be accessible by multi‐step reactions from only one catalysis “world”.

Nature shows in an impressive way that by controlling the microenvironment of catalysts in a defined way, the combination of different reactions is possible. These successful strategies for controlling the microenvironment comprise compartmentalization and scaffolding. In case of (chemo)enzymatic cascade reactions, manifold solutions to tackle compatibility problems have been employed, ranging from compartmentalization of multienzyme complexes in biomembranes, protein nanocages as host for different catalysts, or scaffolding by physical coupling of enzymes.^[19]^ In the field of photocatalysis, the combination of photocatalysts with a diverse range of other catalysts has been systematically investigated. Among others,[^20^, ^21^, ^22^, ^23^, ^24^, ^25^, ^26^] strategies for dual catalytic systems combining homogeneous gold catalysis with visible light photoredox catalysis^[27]^ as well as tandem catalysis employing an anthraquinone‐catalyzed thermal indole‐C3 alkylation in combination with a visible‐light‐driven catalytic photooxidation/1,2‐shift reaction have been developed.^[28]^ Thus, it is not surprising that also the number of examples showing the combination of photo‐ and biocatalysis is rapidly increasing. The probably most common approach yet for the combination of photo‐ and biocatalysis is the photocatalytic in situ regeneration of redox enzymes.[^18^, ^29^, ^30^, ^31^, ^32^, ^33^, ^34^] Hereby, light is used indirectly to fuel chemical transformations, similar to nature's photosynthesis whereby light is used to provide electrons, or to supply a stoichiometric amount of redox equivalents such as NAD(P)H or hydrogen peroxide, which are in turn used for the enzymatic reaction step.[^31^, ^35^, ^36^]

In contrast, the combination of photo(organo)catalytic reactions which use light to directly drive small molecule interconversions with further enzymatic functionalization steps in photo‐biocatalytic one‐pot (tandem) reactions became an emerging field very recently, and a number of examples have been developed in the past three years. Due to the rapid advances in this field and the increasing number of recent achievements, this review summarizes current synthetic examples of PCE cascade reactions and highlights strategies on how compatibility challenges have been tackled. We will first highlight general concepts in photo‐biocatalysis followed by various examples of PCE cascades and will further discuss the scope and limitations of light‐driven approaches with particular emphasis on the reproducibility of photo‐biocatalytic reactions and their future potential for being applied in an industrial setting.

## 2 General Concepts in Photo‐biocatalysis

In search of an efficient strategy to utilize the potential of light to drive biocatalytic conversions, promising basic concepts have been emerged fundamental for a meaningful implementation of whole‐cell as well as *in vitro* photo‐biocatalytic applications.[^30^, ^31^, ^33^] In particular, approaches aiming at the photoactivation of redox enzymes gained increasing attention as strategies to push conventional organic synthesis to an innovative, more sustainable level. The feasibility of biotransformations utilizing the inherent reactivity of natural photoenzymes or photoinduced enzyme promiscuity of cofactor‐dependent enzymes has been explored.^[18]^ The following section provides an overview of general concepts currently developed and applied in the field of photo‐biocatalysis.

### 2.1 Photoactivation of redox enzymes

**Scheme 1:**
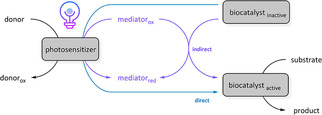
Direct and indirect photoactivation of oxidoreductases by an artificial light‐induced electron transfer.

Owing to their extensive reaction scope, proteins assigned to the class of oxidoreductases, which comprise about 25 % of all enzymes (BRENDA enzyme database), are considered as powerful biocatalysts in the production of pharmaceuticals or fine chemicals.^[37]^ Besides performing enzymatic reductions, the regio‐ and stereoselective oxidation via either dehydrogenation or oxyfunctionalization is of particular interest in organic synthesis.^[38]^ In principle, these reactions are catalyzed by a mediated transfer of electrons or reducing equivalents between a reductant and an acceptor molecule within the active site of the respective enzymes. While in living systems, a continual exchange of reducing equivalents is governed by the metabolic maintenance of a stable and sufficient supply of redox cofactors (e. g., NAD(P)H) or cosubstrates, alternative strategies are inevitable for realizing a rational implementation of redox enzymes in biocatalytic applications.^[39]^ Although enzyme‐coupled cofactor regeneration has become a successful common strategy that is routinely used in industrial applications, the demand for more economic concepts to drive cofactor recycling accompanied by improved atom efficiency, increased significantly. Besides enzymatic, whole‐cell, chemical, and electrochemical cofactor regeneration approaches,[^40^, ^41^, ^42^, ^43^] photochemical concepts inspired by the natural photosystems of photoautotrophs have been developed.[^18^, ^44^, ^45^] As reported in related proof of concept studies, enzymatic redox reactions can be fueled by an artificial light‐mediated transfer of photoexcited electrons or reducing equivalents from a donor substrate towards the redox center of the desired biocatalyst. Whether this transfer shows the need for additional mediator molecules or not, it can be distinguished between indirect or direct enzyme regeneration, respectively (Scheme 1).^[31]^

However, these approaches commonly rely on the presence of photoactive molecules, so‐called photosensitizers, which, unlike photocatalysts, are not directly involved in the bioconversion of a substrate into a corresponding product.^[46]^ Instead, a photosensitizer, in combination with a suitable electron donor as a quencher molecule, can use light to generate photoexcited electrons accessible for various redox biocatalysts. Therefore, porphyrins,^[47]^ flavins,^[48]^ organic dyes,^[49]^ organometallic complexes,^[50]^ or semiconductor‐based quantum dots (QDs)^[51]^ have been successfully applied as photosensitizers to fuel enzymatic redox reactions. As in natural photosynthesis, the use of abundant water as an ideal sacrificial electron donor has major advantages in terms of costs and atom efficiency. However, the potential of H_2_O for the purpose of realizing photo‐biocatalytic approaches is limited due to high stability as well as unfavorable redox potential.^[52]^ Based on that, alternative reductants such as tertiary amines including triethylamine (TEA),^[53]^ triethanolamine (TEOA),[^54^, ^55^] or ethylenediaminetetraacetic acid (EDTA)[^45^, ^48^, ^56^] are commonly used in photo‐biocatalytic reaction systems.^[30]^ Furthermore, the promising dual function of redox‐active buffer agents such as 3‐(*N*‐morpholino)propanesulfonic acid (MOPS), 4‐(2‐hydroxyethyl)‐1‐piperazineethanesulfonic acid (HEPES) or 2‐(*N*‐morpholino)ethanesulfonic acid (MES) as sacrificial electron donors while keeping the pH value at a constant level has been reported in the literature.[^33^, ^35^]

### 2.2 Indirect photoactivation of redox enzymes

Given the fact that the activities of more than 80 % of oxidoreductases are dependent on NAD(P)H, much effort has been made to overcome the need for stoichiometric amounts of expensive and physically unstable nicotinamide cofactors to perform enzymatic redox reactions *in vitro*.[^40^, ^41^, ^42^] While the oxidative potential of NAD(P)^+^ is utilized in enzymatic oxidations via selective dehydrogenation, reduced NAD(P)H function as electron donor providing two electrons and one proton as hydride anion (H^−^) for the corresponding reductive back reactions.^[57]^ Unlike various dehydrogenases (e. g., alcohol dehydrogenase (ADH), aldehyde dehydrogenase) or ene‐reductases (EREDs), oxygenases such as heme‐containing cytochromes P450 (CYPs), flavin‐dependent Baeyer‐Villiger monooxygenases (BVMOs) or Rieske non‐heme iron oxygenases (ROs) are capable of catalyzing oxyfunctionalization via reductive activation and subsequent electrophilic incorporation of oxygen into C−H, C−C as well as C=C double bonds fueled by electrons derived from NAD(P)H.^[38]^

**Scheme 2:**
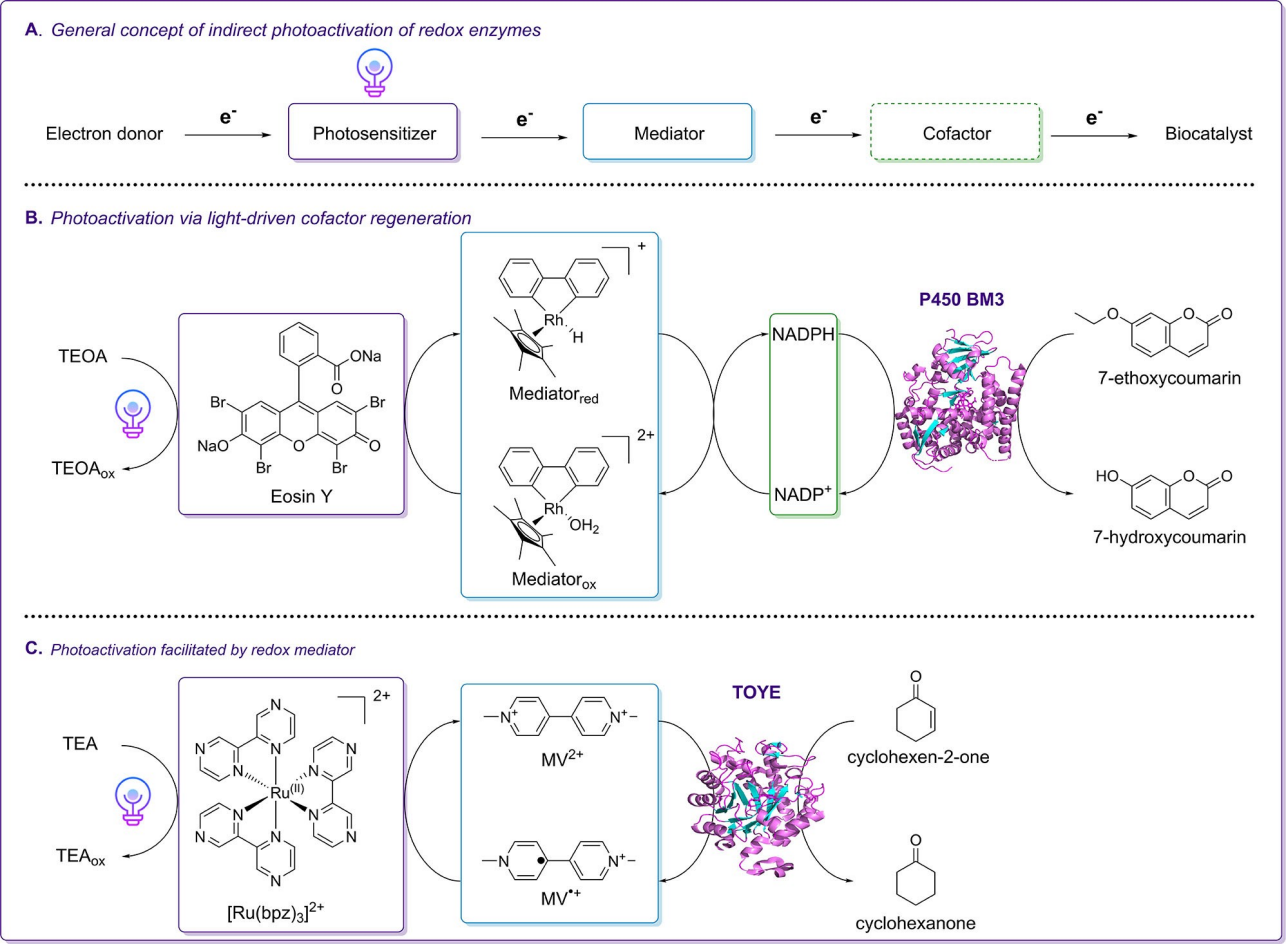
Schematic overview of photo‐biocatalytic concepts applied for the indirect activation of oxidoreductases. A) Simplified representation of the artificial electron‐transfer pathway used for the indirect photoactivation of oxidoreductases. B) Light‐driven approach for the activation of cytochrome P450 BM3 catalyzing O‐dealkylation of 7‐ethoxycoumarin as reported by Lee at al.^[55]^ In the reaction scheme, the xanthene dye eosin Y serves as photosensitizer transferring photoexcited electrons from TEOA to an organometallic mediator molecule facilitating the regeneration of NADPH. C) Enzymatic reduction of an α,β‐unsaturated substrate catalyzed by a thermophilic ene‐reductase (TOYE) fueled by photoexcited electrons derived from the sacrificial electron donor TEA by the use of Ru^II^ based photosensitizer and methyl viologen (MV^2+^) as mediator.^[53]^

Due to this reaction mechanism, new functional groups can be generated, which is highly beneficial for applications in organic synthesis.^[38]^ While only a few studies reported photoinduced regeneration of NAD(P)^+^ as an alternative to commonly applied electrochemical or enzyme coupled approaches, much effort has been made to implement strategies which either aim to perform light‐driven recycling of reduced redox cofactors or even to circumvent their need in *in vitro* biocatalytic applications, respectively.^[31]^ Several studies emphasize the feasibility of artificial light‐mediated electron transfer systems enabling the reduction of NAD(P)^+^ by photoexcited electrons generated by light irradiation of a photosensitizer in the presence of a suitable electron donor (Scheme 2A). To avoid the unfavorable isomerization or dimerization of nicotinamide cofactors,^[42]^ electron mediators such as organometallic [Cp*Rh(bpy)H_2_O]^2+^ are commonly applied and are highly selective for the generation of the active 1,4‐cofactor variants.[^30^, ^31^, ^54^] Photoregeneration has been successfully coupled to the catalytic activity of glutamate dehydrogenase, formate dehydrogenase or CYPs using TEOA as a sacrificial electron donor in combination with proflavin,^[54]^ QDs,^[51]^ graphene‐based photosensitizers^[44]^ or organic dyes^[55]^ as photosensitizers (Scheme 2B). Analogously, electron mediators such as methyl viologen (MV) or flavins have been successfully used for the photoregeneration of various NADPH‐ and flavin‐dependent oxidoreductases. One example is the light‐driven conversion of cyclohexen‐2‐one catalyzed by TOYE (Scheme 2C).^[53]^ Besides transferring reducing equivalents between a photosensitizer and a redox biocatalyst in homogeneous reaction systems, transfer properties of electron mediators have been exploited in photo‐biocatalytic applications via photoelectrochemical (PEC) cell platforms. In these concepts, (photo)electrodes are used to activate oxidoreductases by regenerating the required redox cofactors as well as to transfer photoexcited electrons to the enzyme's active site.^[58]^

While biocatalysts assigned as oxygenases show the need for reduced redox cofactors delivered by an electron transport chain to activate molecular O_2_, enzymes belonging to the group of peroxygenases are capable of directly using H_2_O_2_ as a cosubstrate for the oxyfunctionalization of C−H bonds.^[59]^ However, due to their sensitivity towards H_2_O_2_, their implementation in large‐scale applications is still challenging.[^59^, ^60^] Recent studies proposed the light‐driven in situ generation of H_2_O_2_ as a promising approach[^36^, ^61^, ^62^, ^63^, ^64^, ^65^] exhibiting improved atom efficiency compared to the conventional enzyme‐catalyzed cosubstrate supply.^[66]^ Thereby, light exposure of organic or TiO_2_‐based photocatalysts in the presence of a suitable electron donor yields in the formation of photoexcited reducing equivalents, capable of the subsequent reduction of O_2_ to the stoichiometric oxidant H_2_O_2_. Thus, a constant low level of H_2_O_2_ can be maintained proven to have a positive impact on the operational enzyme stability of various peroxygenases.[^36^, ^62^, ^67^]

### 2.3 Direct photoactivation of redox enzymes

Besides the activation of oxidoreductases by mediators functioning as relay system, several studies proposed the implementation of simplified reaction systems based on direct interactions between photosensitizers and redox centers of the respective enzymes. By that, the need for stoichiometric amounts of cofactors and the disadvantageous properties of mediator molecules can be circumvented. (Semi)homogeneous reaction systems based on a direct transfer of photoexcited electrons from organic photosensitizers,[^48^, ^68^, ^69^] TiO_2_ semiconductor‐based materials,^[52]^ or carbon nanodots^[70]^ to flavin‐dependent enzymes or hydrogenases have been reported in the literature. Furthermore, protein hybrid systems composed of oxidoreductases attached to the surface of semiconductor materials such as TiO_2_ ^[71]^ or Cd‐based particles[^72^, ^73^, ^74^] as well as functionalized carbon dots^[75]^ have been applied. Driven by light exposure of hybrid photocatalysts in the presence of sacrificial electron donors in solution, direct photoactivation of various metalloenzymes could be induced, performing bioconversions such as CO_2_ ^[71]^ or N_2_ ^[74]^ reduction and H_2_ generation.[^72^, ^73^]

**Scheme 3:**
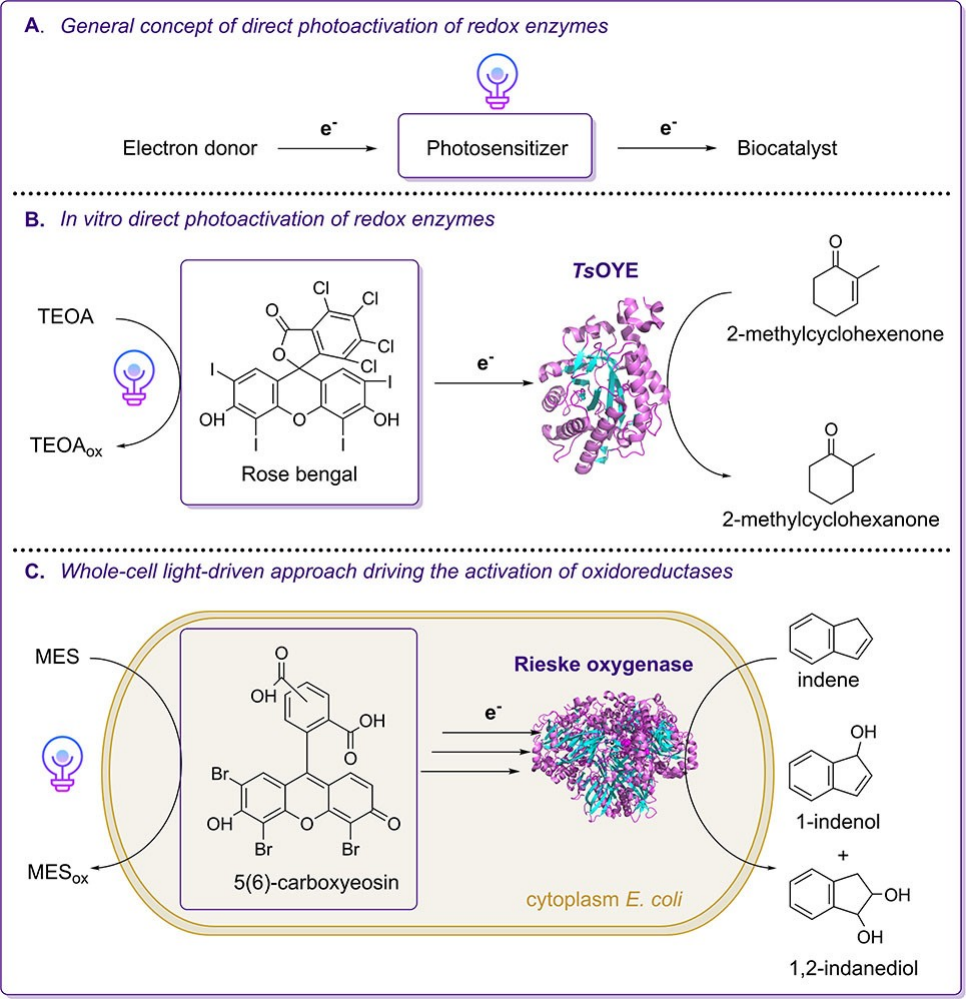
Schematic overview of strategies for the direct photoactivation of redox biocatalysts. A) Schematic representation showing the proposed electron‐transfer chain established in approaches aiming at the direct photoactivation of oxidoreductases. B) *In vitro* light‐driven asymmetric reduction of C=C bonds catalyzed by an OYE variant from *Thermus scotoductus* (TsOYE).^[69]^ As reported by the authors, rose bengal is a suitable photosensitizer for transferring photoexcited electrons derived from TEOA towards the prosthetic FMN group of TsOYE. C) Light‐driven whole‐cell approach using resting *E. coli* cells harboring a Rieske oxygenase (RO) as biocatalyst.^[45]^ MES buffer serves as electron donor for 5(6)‐carboxyeosin (photosensitizer), which initiates the transfer of photoexcited reducing equivalents to the non‐heme iron center of the terminal RO.^[45]^

Moreover, the direct covalent linkage of photosensitizers to the prosthetic group of oxidoreductases has been demonstrated for CYPs using Ru^II^‐diimine for photosensitization.^[76]^ Lee et al. described the direct photoregeneration of OYE homologs catalyzing the stereoselective reduction of C=C bonds.^[69]^ Thereby, TEOA as electron donor was used in combination with soluble xanthene dye derivates, which functioned as photosensitizers (Scheme 3B).^[69]^ Free flavins can act as photosensitizers as well as cofactor capable of reducing the prosthetic group of flavoenzymes such as in BVMOs or EREDs in the presence of sacrificial electron donors under light exposure.[^48^, ^68^] Analogously to cell‐free light‐driven regeneration systems, recent proof of concept studies proposed whole‐cell approaches taking advantage of the preserved cytosolic environment in non‐autotrophic microorganisms without interfering with their natural metabolism. As proposed, oxyfunctionalization initiated by the light‐mediated transfer of photoexcited electrons from biocompatible photosensitizers to the prosthetic group of CYPs,^[77]^ ROs^[45]^ and a hydrogenase^[78]^ in *E. coli* applied as whole‐cell biocatalysts was successfully shown (Scheme 3C).

### 2.4 *In vivo* light‐driven cofactor regeneration

**Scheme 4:**
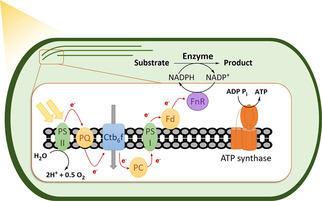
Schematic representation of *in vivo* light‐driven cofactor regeneration by exploiting the natural photosystems involved in the oxygenic photosynthesis of photoautotrophs coupled with heterologous oxidoreductases. Electrons generated by photoinduced water splitting in photosystem II (PSII) are shuttled successively via a cytochrome b6 f complex (Ctb6 f) and photosystem I (PSI) to a terminal reductase (FnR), which catalyzes the regeneration of NADPH. PQ: plastoquinone, PC: plastocyanine, Fd: ferredoxin.

Besides linking heterotrophic cells to light‐driven regeneration approaches, autotrophic organisms such as cyanobacteria recently received attention due to their ability to utilize inorganic compounds as electron donors.[^79^, ^80^, ^81^, ^82^] By implementing exogeneous biochemical pathways and metabolic engineering strategies, cyanobacteria have been proposed as potential platform organisms for the production of biofuels such as ethanol, butane‐2,3‐diol, or isopropanol from CO_2_.[^83^, ^84^] Furthermore, it has been shown that heterologous enzymes such as CYPs,^[82]^ imine reductases,^[85]^ alkane monooxygenases,^[81]^ EREDs^[80]^ or BVMOs^[79]^ can be coupled successfully to photosynthetic NADPH generation (Scheme 4). Various oxyfunctionalization reactions, imine reductions as well as asymmetric reductions of C=C bonds could thus be performed in recombinant cyanobacterial strains such as *Synechocystis* sp. PCC 6803.

### 2.5 Photoenzymes for photo‐biocatalytic applications

**Scheme 5:**
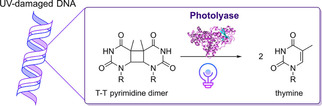
Photoreactivation of a T−T pyrimidine dimer in UV‐damaged DNA catalyzed by a photolyase.^[86]^

Besides biocatalytic concepts exploiting the light‐harvesting properties of photosystems from photoautotrophs to drive light‐driven biotransformations, a reasonable implementation of the inherent reactivity of enzymes referred to as “photoenzymes” capable of the direct conversion of light into chemical energy has been investigated. However, the scope of available photoenzymes is yet very limited, and their currently evaluated biocatalytic applicability is primarily restricted to their naturally substrate specificity. Flavoproteins assigned to the class of photolyases play a crucial role in the elimination of pyrimidine dimers that emerged by UV‐induced DNA damage (Scheme 5).^[86]^ Despite catalyzing DNA photoreactivation at considerable high efficiency, an expedient implementation of photolyases in biocatalytic approaches has not been described in literature yet.^[87]^

**Scheme 6:**
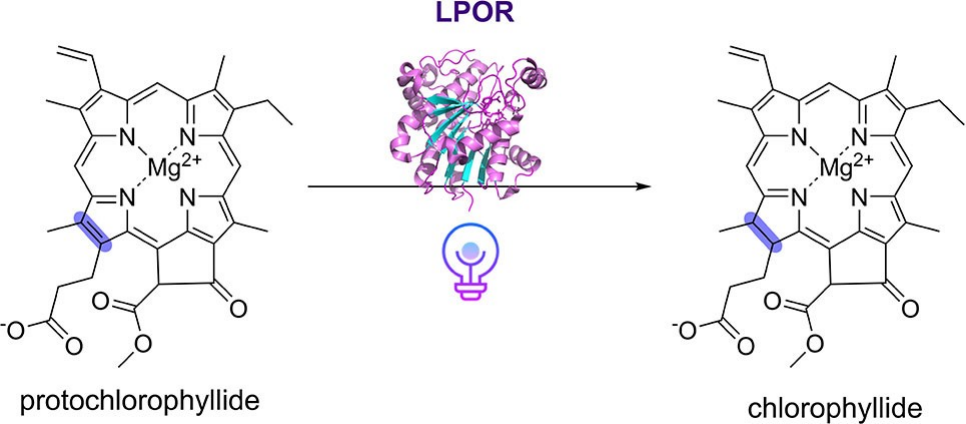
Photoinduced reduction of protochlorophyllide to chlorophyllide catalyzed by a light‐dependent protochlorophyllide reductase (LPOR).^[88]^

Based on previous findings, light‐dependent protochlorophyllide reductases (LPORs) are involved in the biosynthesis of chlorophyll in oxygenic as well as anoxygenic phototrophs catalyzing the reduction of protochlorophyllide (pchlide) into chlorophyllide (chlide, Scheme 6).^[88]^ Although *in vitro* assays for LPORs in the presence of NADPH under light exposure yielded in the conversion of various pchlide derivates into the corresponding reduced products, applications beyond the bioconversion of natural substrates have not been reported yet.^[89]^

Several LPORs from different origins were biochemically characterized and applied in the light‐driven conversion of pchlide to chlide under different conditions (light intensity, solvent content, pH range) and were analyzed for their cofactor flexibility.^[90]^

Recently, a fatty acid photodecarboxylase (FAP) from microalgae has gained increasing attention due to their ability to catalyze a light‐mediated decarboxylation of fatty acids,^[91]^ which may be applied for sustainable biofuel production from natural resources.^[92]^ Under blue light irradiation, *Cv*FAP from *Chlorella variabilis* converted long‐chain fatty acids into the corresponding alkanes with highly promising turnover numbers and almost full conversion.^[93]^ Engineered *Cv*FAP variants catalyzing the selective decarboxylation of α‐functionalized carboxylic acids have been generated and may be promising catalysts for the light‐driven cofactor‐independent kinetic resolution of various αhydroxy amino acids.^[94]^ Recently, enzymatic cascades combining the activity of lipases with the reactivity of *Cv*FAPs have been developed. By that, the successive conversion of various triglycerides from natural oils into the respective alkanes was demonstrated (see also Section 3.2).^[92][95]^

### 2.6 Light‐induced enzyme promiscuity

**Scheme 7:**
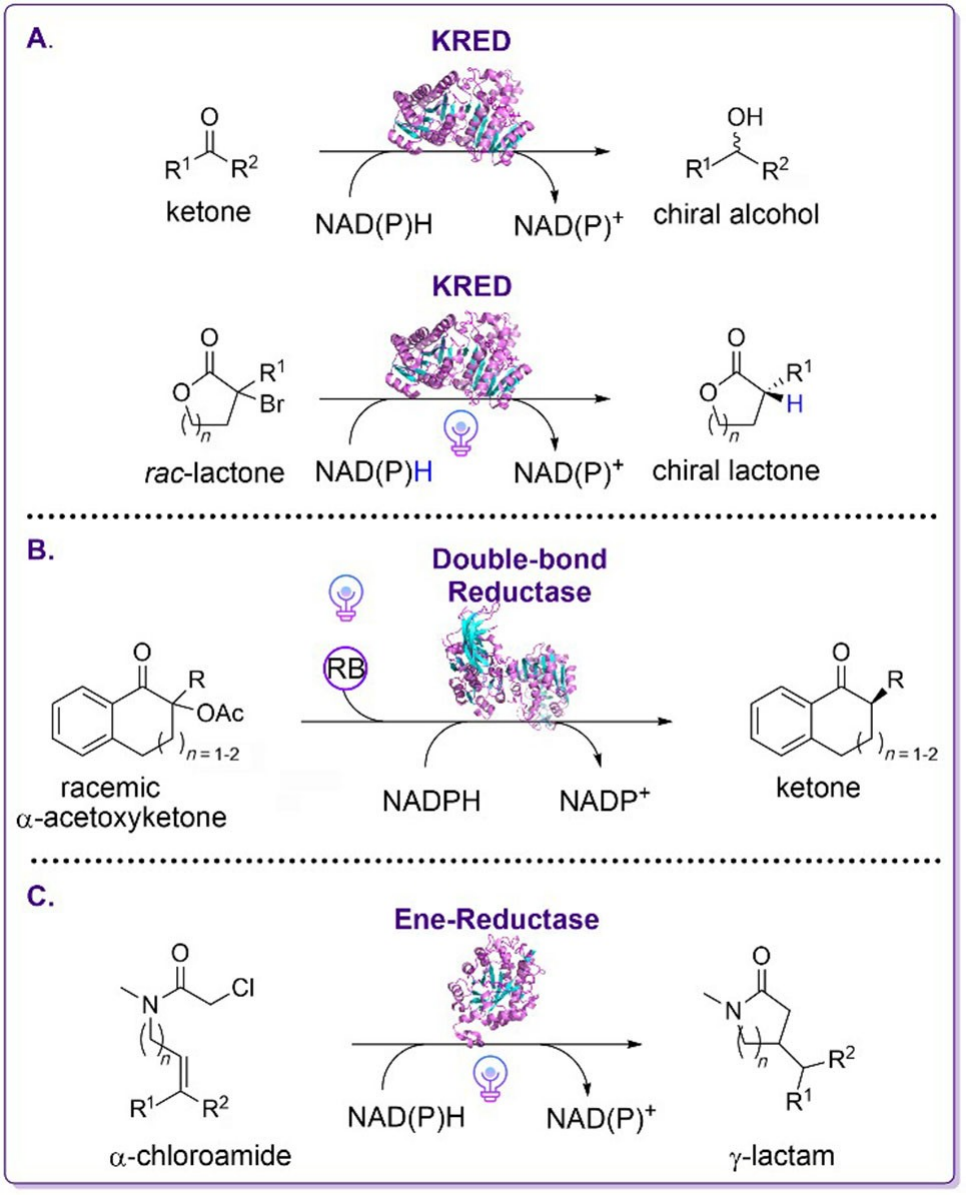
Schematic overview of light‐driven biocatalytic approaches exploiting photoinduced promiscuity of NAD(P)H‐dependent enzymes and flavoenzymes. A) Top: Natural reduction of ketone substrates into chiral alcohols catalyzed by KREDs.^[96]^ Bottom: Unnatural photoinduced activity of KREDs catalyzing enantioselective dehalogenation of halolactone substrates.^[96]^ B) Altered enzyme activity of double‐bond reductases catalyzing the deacetoxylation of α‐acetoxyketones by using photoexcited rose bengal (RB).^[97]^ C) Radical cyclization of α‐chloroamide substrates into γ‐lactams by photoexcitation of flavin‐dependent EREDs.^[98]^

Tailoring the catalytic properties of enzymes by directed evolution or rational design is crucial for their efficient implementation in industrially relevant processes. However, recent studies reported catalytic promiscuity of various oxidoreductases driven by the photoexcitation of NAD(P)H or flavin cofactors as well as organic photosensitizer yielding in the generation of radical intermediates accessible for biocatalytic reactions. By that, a range of unnatural reactions can be performed, circumventing the need for enzyme engineering techniques.[^96^, ^97^, ^98^] A study conducted by Emmanuel at al. confirmed the light‐mediated alteration of the natural enzyme activity of ketoreductases (KREDs) towards enantioselective dehalogenation of halolactones (Scheme 7A).^[96]^ The authors concluded that the respective radical reaction mechanism is initiated by photoexcitation of NAD(P)H as well as halolactones in the active site of the KREDs (charge‐transfer complex, CT complex), generating the corresponding cofactor and substrate radicals. The catalytic cycle is completed by mesolytic cleavage of the C−X bond followed by a hydrogen transfer from NAD(P)H to the lactone yielding in the dehalogenated product.^[96]^

Hyster and co‐workers reported an enantioselective deacetoxylation of α‐acetoxyketone substrates by an unnatural reactivity of double‐bond reductases utilizing the photoexcitability of organic photosensitizers (Scheme 7B).^[97]^ It is proposed that green‐light irradiation of rose bengal (RB) in the presence of NAD(P)H yields in the formation of radical RB^.−^. This intermediate is then capable of reducing the enzyme‐bond substrate yielding in the formation of a deacetoxylated α‐acyl radical. In the final step, hydrogen transfer from reduced NADPH yields in the respective product.^[97]^ Besides the described photoinduced promiscuity of NAD(P)H‐dependent enzymes, non‐natural reactivity of flavin‐dependent EREDs has been observed, which is initiated by light‐mediated radical reaction mechanisms.^[98]^ Thereby, the formation of the corresponding lactam products is achieved by the photoexcitation of the CT complex formed by reduced flavin cofactor and α‐chloroamide substrates within the active site of the respective enzyme (Scheme 7C).^[98]^

**Scheme 8:**
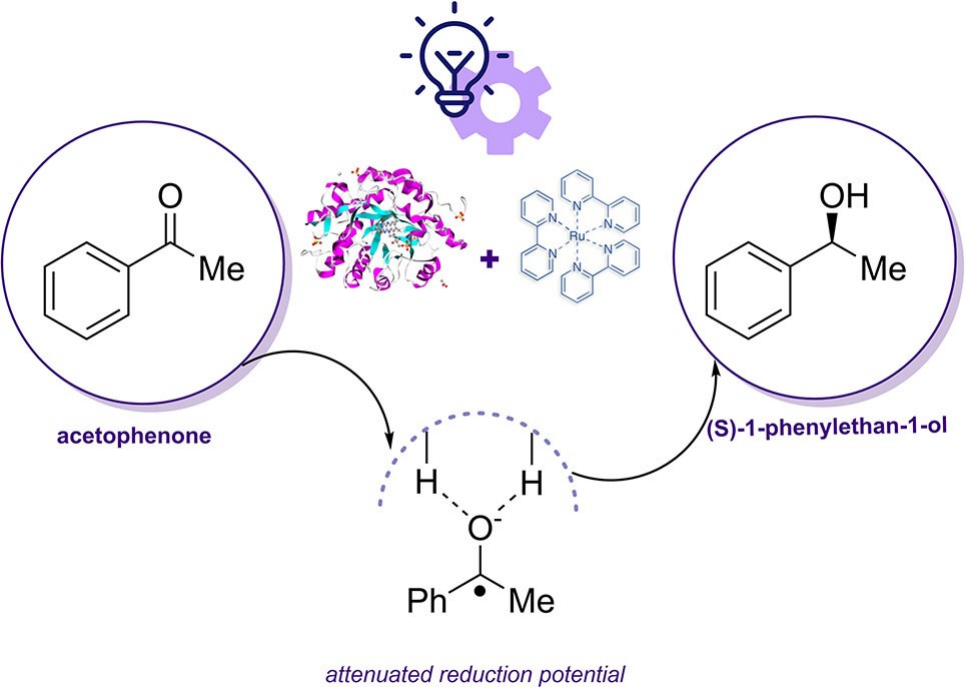
Schematic representation of the photo‐redoxcatalytic cascade reaction mediated by the combination of [Ru(bpy)_3_]Cl_2_ coupled to Flavin‐dependent EREDs.^[99]^

In a follow‐up work, the same group demonstrated that EREDs could catalyze the reduction of carbonyls in the presence of photoredox catalysts, a reaction that goes beyond the usual reactivity of EREDs.^[99]^ The proof of concept was performed with acetophenone as model substrate and [Ru(bpy)_3_]Cl_2_ as photocatalyst. When the most active enzyme, morphine reductase (MorB), was used, the corresponding alcohol was obtained with 99 % yield favoring the *R* enantiomer (80 : 20 *er*, Scheme 8).

Electron transfer through photoexcitation of a CT complex can facilitate the catalytic reactions of unstabilized radicals under visible light irradiation as reported in previous studies. Based on this perception, Clayman and Hyster conducted a prospective study showing the improved accessibility of enantioselective radical cyclization using alkyl iodides as a precursor for the generation of a nucleophilic radical.^[100]^ The hypothesis was then developed to address several strategies to incorporate the advantages of photoenzymatic radical cyclization of alkyl iodide to chiral lactone catalyzed by EREDs. Thereby, considerable product yields of up to 97 % and promising enantioselectivities could be achieved in the conversion of various carbonyl substrates. To substantiate the proposed electron transfer mechanism facilitated by the CT complex, isotopic labeling experiments were performed confirming the generation of α‐acyl radicals generated by hydrogen atom transfer (HAT) mechanisms from flavin. Consequently, the scope of the concept shows that simple esters as well as carbonyl compounds, other alkyl radical precursors are effective and compatible substrates of these systems for their application in (chemo)enzymatic cascade reactions.

## 3 Cascades Combining Photo‐Chemocatalytic and Biocatalytic Transformations

Nowadays, the importance of cascade reactions in biotechnological applications is rapidly growing. The addition of all catalysts and solvents at once makes one‐pot linear cascades (referred to as concurrent or tandem reactions) much more preferable due to the direct consumption of intermediates in the subsequent reaction step, resulting in the desired product without intermediate work‐up.^[3]^ Light is one of the most important alternative sources to drive PCE cascade reactions. In recent studies, the use of light has been considerably increased in order to extend the photo‐biocatalytic toolbox for the synthesis of various pharmaceutical and fine chemicals. The application of a photocatalyst in one‐pot reactions that are performed either simultaneously or sequentially is feasible. However, the decision whether a PCE reaction is performed sequentially or simultaneously strongly depends on the type of photocatalyst, possible side reactions, and the stability of the enzyme in the presence of the photocatalyst. The functional combination of the photocatalytic reaction with an enzyme may allow the design of powerful biotransformations utilizing the reactivity of the photocatalysts in combination with the effectiveness of enzymes in terms of selectivity and activity.^[101]^ However, there are critical challenges that need to be overcome in order to run such systems with high efficiency. In the following, we will highlight several newly developed PCE cascades and approaches that have been applied in order to overcome limitations that were faced.

### 3.1 Simultaneous and sequential photo‐chemoenzymatic cascades

**Scheme 9:**
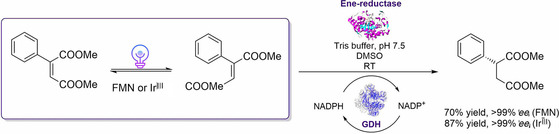
Concurrent photo‐chemoenzymatic approach combining a photocatalytic isomerization and the enzymatic reduction of alkenes. GDH: glucose dehydrogenase, NADP(H): (reduced) nicotinamide adenine dinucleotide phosphate, RT: room temperature.^[101]^

Concurrent PCE cascade reactions are generally difficult to establish due to the often opposite reaction conditions that a photocatalyst and an enzyme require (both need to operate in aqueous solution and at ambient temperature) and stability problems that may arise in the presence of the enzyme, substrate and product in the reaction mixture. Hartwig and colleagues recently showed the compatibility of a photocatalyst isomerizing alkenes with EREDs in a PCE reaction for C=C‐bond reductions to generate valuable enantio‐enriched products.^[101]^ After screening a number of organometallic and organic photocatalysts in semi‐aqueous medium, the simultaneous, cooperative photoisomerization and enzymatic reduction of the *Z*‐configured substrates were investigated. For a range of different substrates, the cooperative reduction catalyzed by different EREDs and different photocatalysts such as FMN and Ir^III^ under blue light illumination could be achieved with product yields of up to 87 % and an *ee* of >99 % (Scheme 9). The authors emphasized that in general, two features of photocatalysts make them suitable for PCE cascades. First, the photocatalytic reaction must occur at or at least near room temperature in order to match the enzyme‘s requirements, and second, the mechanism underlying the photocatalytic reaction must involve intermediates that are stable towards water and functional groups in proteins.

**Scheme 10:**
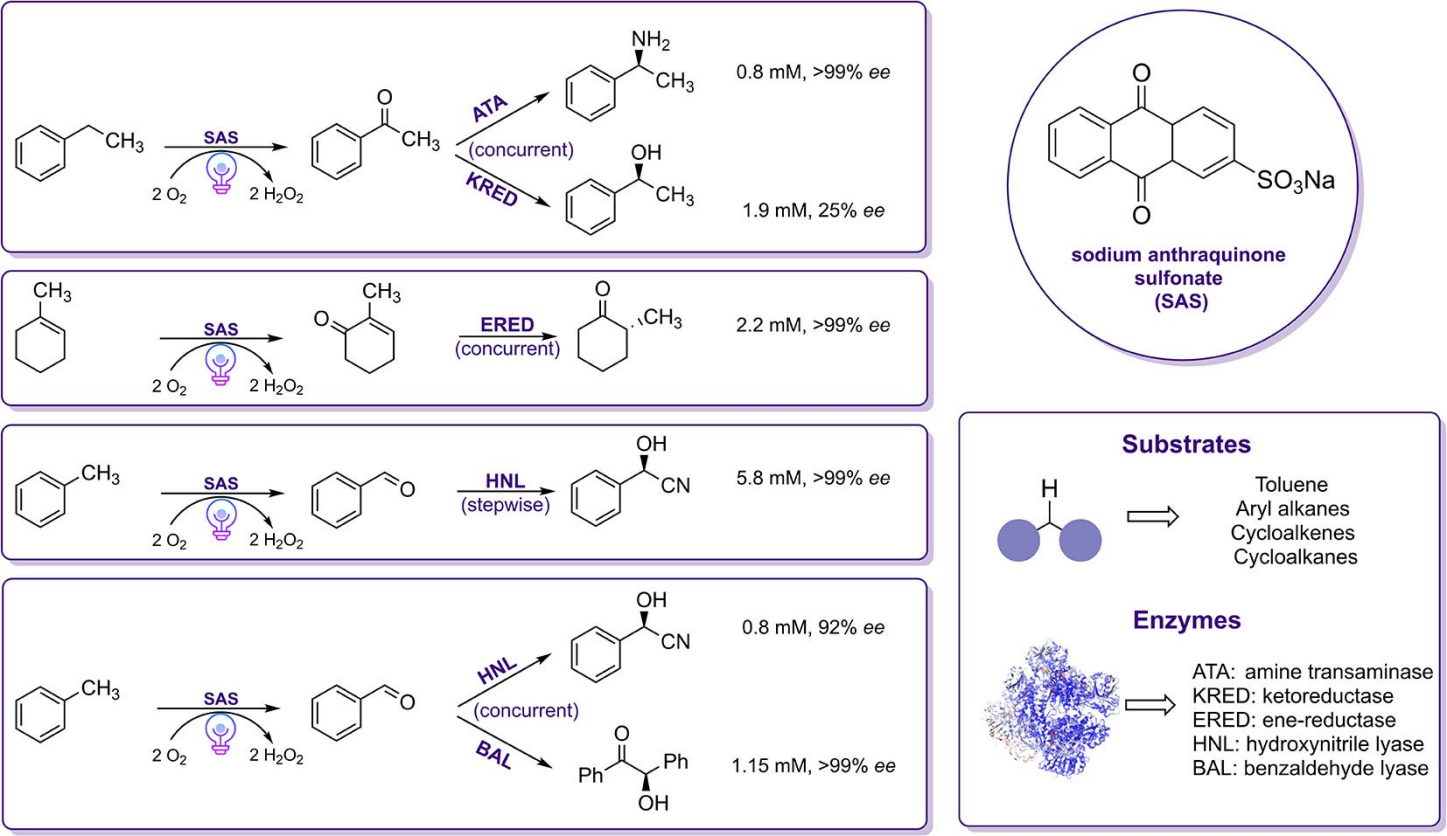
Photo‐enzymatic cascade reaction for C−H functionalization by combining SAS as photocatalysts with different enzymes. The first step is performed as a two‐phase reaction. Reaction conditions light: substrate (20 mM), 10 % (*v*/*v*) acetonitrile in aqueous reaction buffer, 1–2 mg/mL enzyme (lyophilisate), at 30 °C, for 6–24 h under white light illumination.^[102]^

Another example shows that combining the advantages of photo‐organo‐redox catalysis with the activity of biocatalysts can be used for the asymmetric C−H oxyfunctionalization of various alkanes converting them to the corresponding aldehydes or ketones.^[102]^ The photocatalyst sodium anthraquinone sulfonate (SAS) was used for the initial alkane oxidation step. This step was then followed by the enzymatic transformation of the intermediary aldehyde/ketone. Several enzymes have been investigated in order to obtain the desired high‐value products, such as formic esters, lactones, chiral cyanohydrins, chiral acyloins, carboxylic acids, chiral cyclohexanones, and amines. The successful implementation of this PCE cascade has been demonstrated by the synthesis of several products, which indicates the synthetic value of this approach (Scheme 10). Numerous chiral products covering a wide range of functional groups have been obtained by using simple and cheap alkanes as starting material. Schmidt and co‐workers synthesized (*R*)‐benzoin and (*R*)‐mandelonitriles at gram‐scale with high isolated yields and excellent *ee* (>99 %). Although these two examples were successfully demonstrated on a semi‐preparative scale, further optimization of other cascade reactions was necessary in order to solve the problem of catalyst inhibition/deactivation due to the formation of reactive oxygen or radical species or cross‐reactivities in the presence of light.

Even though amine transaminases (ATAs) were active in the presence of DMSO, the co‐solvent led to a decrease in the photocatalytic activity of SAS by 25‐fold. The inactivation of the catalyst that occurred due to the applied co‐solvent was hindered by establishing a two‐phase reaction system. The same strategy has been followed for the KRED reaction, in which the alcohol products were protected for facile oxidation by a two‐phase approach, thus increased the obtained product concentrations by tenfold. To avoid such incompatibilities of reaction conditions, the group conducted the cascade sequentially by adding the appropriate biocatalyst after the photocatalytic step and achieved a sixfold increase in product formation up to 5.8 mM (*R*)‐mandelonitrile with an enantiomeric excess of 99 % *ee* in the hydroxynitrile lyase‐catalyzed reaction. Based on the obtained results, incompatibility between enzyme and photocatalyst can be solved by a two‐phase approach or by spatial or temporal separation of the photo(chemo)‐ and biocatalytic reaction step. In addition, protein engineering, as pointed by the authors, can be an effective tool for developing enzymes with improved activities and stabilities in order to further increase catalyst compatibility in photo‐biocatalytic cascades.

Volatile sulfur compounds (VSCs) are highly desirable chemicals, which can be used in some industrious ways to produce, for example, valuable flavors, aromas of foods and beverages. Moreover, their olfactory perception is based on their configuration.^[103]^ Among the VSCs, 1,3‐mercaptoalkanols can be obtained in enantiomerically pure form through preparative gas chromatography resolution or ketone reductions. However, these methods impose limitations for the sustainable production with respect to the atom economy, the use of non‐green solvents, and catalyst recyclability. Similar to the work of Schmidt and co‐workers, Lauder et al. described the first one‐pot sequential cascade for the synthesis of enantiomerically pure 1,3‐mercaptoalkanols in a photocatalyzed thio‐Michael reaction and a subsequent biocatalytic ketone reduction of the carbonyl group using two highly selective KREDs with opposite enantioselectivity.^[104]^ The first step of the cascade was carried out using [Ru(bpy)_3_]Cl_2_ as photocatalyst under visible light irradiation.

**Scheme 11:**
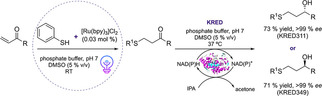
One‐pot photo(chemo)catalytic cascade comprising a thio‐Michael addition and a subsequent KRED‐catalyzed transformation to the desired 1,3‐mercaptoalkanols. IPA: isopropyl alcohol, NADH: nicotinamide adenine dinucleotide, NADPH: nicotinamide adenine dinucleotide phosphate.^[104]^

In order to develop an efficient one‐pot protocol for the synthesis of mercaptoalkanols directly from alkenes, an alternative photocatalytic thiol‐ene approach was investigated. The reaction was performed in a mixture of DMSO and phosphate buffer under visible light irradiation leading to full conversion to the desired ketone intermediate in less than 5 min and the final product, 1,3‐mercaptoalkanols, were subsequently obtained with a yield of ≥70 % and excellent optical purity of >99 % *ee* within 24 h (Scheme 11).

**Scheme 12:**
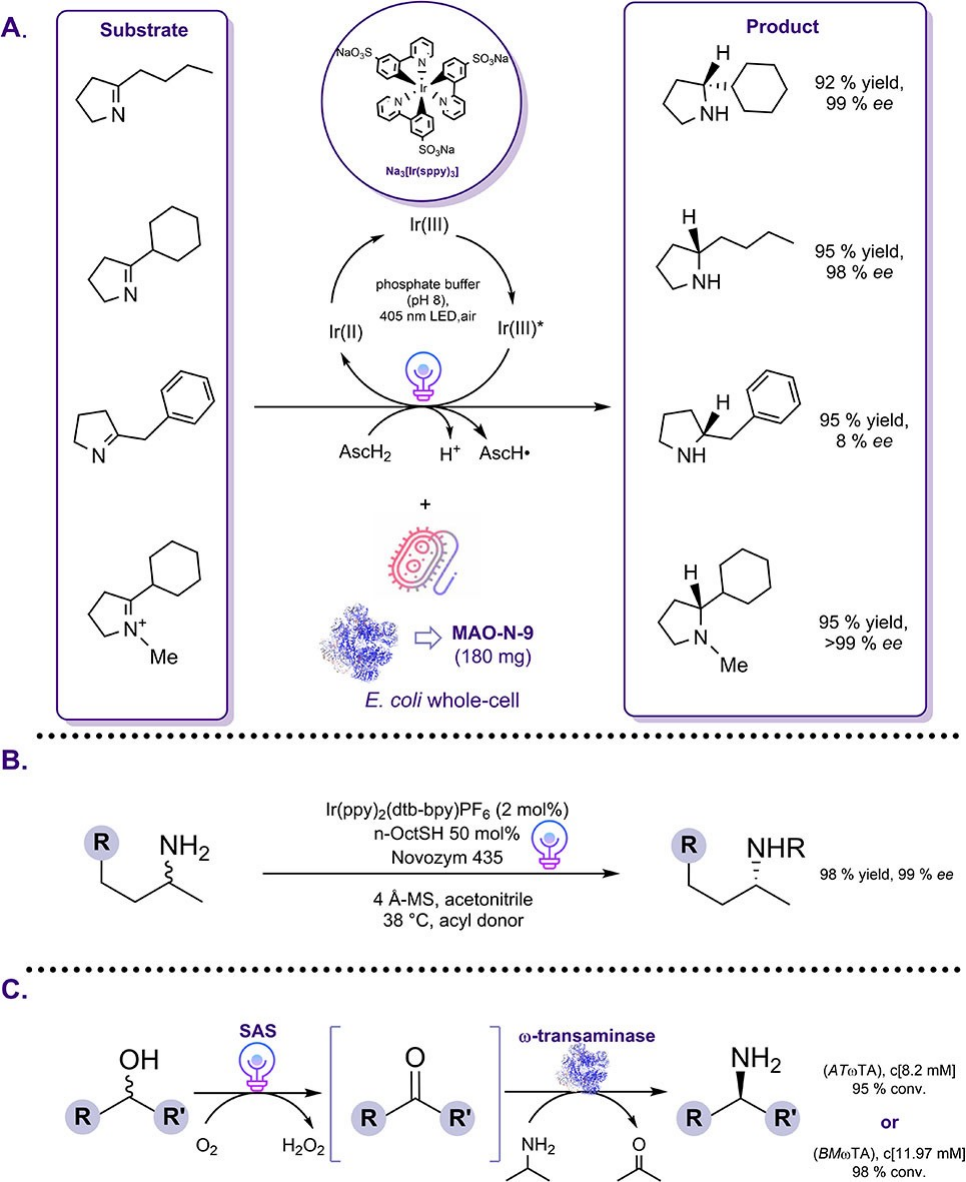
A) Synthesis of amines from imines in a combined photoredox‐enzyme catalysis approach. Reaction conditions: substrate (10 mM), *E. coli* whole cells (MAO‐N‐9; 180 mg, wet weight), photocatalyst (1 mol %), phosphate buffer (pH 8.0), at ambient temperature with 405 nm LED (3 W) irradiation.^[106]^ B) Combination of visible‐light photoredox‐ and enzyme catalysis for the DKR of amines. Reaction conditions: photocatalyst Ir(ppy)_2_(dtb‐bpy)PF_6_ (2 mol %), amine (0.4 mmol), acyl donor (2 equiv.), n‐OctSH (50 mol %), Novozym 435 (80 mg), 4 Å MS (400 mg) at 38 °C under irradiation white LED lamp (32 W).^[109]^ C) Synthesis of enantiomerically pure amines by linking sequentially the photocatalytic oxidation of alcohols with an enantioselective amination of ketones catalyzed by two enantio‐complementary ω‐TAs: *R*‐selective ω‐TA from *Aspergillus terreus* (*AT*ωTA) and the *S*‐selective ω‐TA from *Bacillus megaterium* (*BM*ωTA). Reaction conditions: SAS (0.75 mM), substrate (10 mM), crude cell extract containing ω‐TA (10 mg/mL), IPA (1 M), PLP (1 mM) in phosphate buffer (50 mM). Samples were incubated at 30 °C under visible light illumination.^[111]^

In recent years, photo‐redox chemistry has received considerable attention for the synthesis of amines as an atom‐economic and environmentally benign approach. Enantiomerically pure amines obtained via photoenzymatic cascades can be used as chiral ligands, catalysts, and kinetic resolution reagents in pharmaceutical and natural product synthesis.^[105]^ Along this line, several strategies to improve the photo‐biocatalytic synthesis of chiral amines have been investigated. Wenger and co‐workers developed a cyclic reaction network strategy for the synthesis of enantio‐enriched amines by linking the light‐driven reduction of imines catalyzed by a water‐soluble photocatalyst (Na_3_[Ir(sppy)_3_]) with an enzymatic transformation catalyzed by monoamine oxidase (MAO−N‐9), leading to the corresponding chiral amines (Scheme 12A).^[106]^ The excitation of this water‐soluble photosensitizer in the presence of a cyclic imine affords a highly reactive α‐amino alkyl radical that is intercepted by HAT from ascorbate or thiol donors to afford the corresponding amine. The high reducing power and the solubility in water of Na_3_[Ir(sppy)_3_] enabled the successful conversion of imines to racemic amines in 95 % yield within 10 h with the use of ascorbic acid as a HAT donor upon illumination. In the second step of the cascade, *E. coli* cells containing overexpressed MAO−N‐9 were used for the enantioselective amine oxidation of cyclic amines as illustrated in Scheme 12A. The model substrate, 2‐cyclohexyl‐1‐pyrroline, was converted to the respective pure (*R*)‐amine with >92 % yield and high enantioselectivity with up to 99 % *ee* after prolonged reaction time. Subsequently, the group performed the cascade by employing *E. coli* cell lysates containing MAO−N‐9 and a decrease in conversion (83 %) with lower enantioselectivity (77 % *ee*) was observed. The observed decrease of activity was attributed to partial inactivation of MAO−N‐9 by the photocatalyst. As it was observed that the enzyme‐catalyzed amine oxidation is the rate‐determining step, the low yield might be obtained due to the detrimental effect of the biocatalyst on the photo‐redox process. To extend the applicability of this concept, several imine substrates with aliphatic and aromatic substituents have been explored. Although the substrate scope remained limited to the reduction of imine substrates with phenyl, alkyl or benzyl substituents, the addition of various thiols enabled the expansion of the substrate scope to 1‐methyl‐3,4‐dihydroisoquinoline.^[107]^ In order to enable polarity‐matched catalysis for HAT, the combination of ascorbic acid with aliphatic or aromatic thiol additives, depending on the substrate, is required.

The combination of asymmetric photo‐ with enzyme catalysis in dynamic‐kinetic resolutions (DKR) is an elegant approach for the diastereo‐ and enantioselective synthesis of amines in high yield.^[108]^ However, the DKR of amines has been extremely challenging due to the lack of compatible catalysts for the amine racemization since these catalysts often perform under different conditions. Zhou and co‐workers demonstrated the combination of enzymatic DKR with photo‐redox mediated HAT for the racemization of amines (Scheme 12B).^[109]^ A wide variety of photocatalysts were initially investigated to optimize the racemization conditions. The use of 2 mol % of the photocatalyst Ir(ppy)_2_(dtb‐bpy)PF_6_ together with HAT catalyst *n*‐OctSH under light irradiation gave the best results. For the enzymatic resolution via acylation, *Candida antarctica* lipase B (Novozym 435) was used with methyl β‐methoxypropionate as the acyl donor. Without observing detrimental inactivation effects, a complete conversion of the racemic amine into the corresponding chiral (*R*)‐amide with excellent yield (98 %) and an *ee* of 99 % could be achieved (Scheme 12B).

More recently, MacMillan, Hyster and co‐workers reported a novel DKR approach based on the photoredox catalyzed racemization of static stereocenters of β‐substituted carbonyl compounds.^[110]^ As indicated by the authors, this strategy could be successfully applied for the DKR of various β‐substituted ketones into corresponding chiral alcohols by combining organo‐ and photoredox catalysis, followed by a final stereoselective enzymatic reduction. The proposed photoredox‐catalyzed racemization is initiated by the condensation of *tert*‐butyldiphenylsilyl with the inactive ketone substrate yielding in the formation of a stereo‐defined enamine. In the following light‐driven step, the enamine intermediate is oxidized by the photoexcited [Ir(dF(CF_3_)ppy)_2_(dtbbpy)](PF_6_) photocatalyst resulting in the formation of a prochiral β‐enaminyl radical species accompanied by a loss of the stereochemical properties of the reactant. Hydrogen atom transfer facilitated by 4‐methoxythiolphenol (HAT catalyst) and subsequent hydrolysis resulted in the formation of the desired β‐substituted ketones, which serve as a suitable substrate for the final enzymatic conversion to respective γ‐substituted alcohol. Thereby, product yields of up to 92 % (>99 : 1 *er* and >20 : 1 *dr*) could be obtained with *Lactobacillus kefir* ADH. To further explore the scope of this approach, aminotransferases have been investigated to demonstrate the applicability and biocompatibility of this novel chemoenzymatic DKR protocol that can afford the synthesis of chiral amines in high yields. Based on the obtained results, the feasibility of kinetic resolutions with the racemization strategy will enable the improvement of challenging stereoconvergent syntheses of complex compounds.

Recently, Hollmann and co‐workers developed a one‐pot sequential cascade combining a light‐driven oxyfunctionalization reaction using SAS as photocatalyst with a subsequent biocatalytic reductive amination by using highly selective ω‐transaminases (ω‐TA) from different origins (Scheme 12C).^[111]^ Numerous photocatalysts were initially investigated to devise an optimal model reaction for the conversion of *rac*‐1‐phenylethanol to the corresponding ketone (acetophenone). According to the obtained results, the group focused their attention on SAS and heterogeneous graphitic carbon nitride (g‐C_3_N_4_). In the one‐pot one‐step cascade, two ω‐TAs were preferred for the reductive amination: the *R*‐selective ω‐TA from *Aspergillus terreus* (*AT*ωTA) and the *S*‐selective ω‐TA from *Bacillus megaterium* (*BM*ωTA). It was observed that the oxidation activity of g‐C_3_N_4_ was reduced due to the absorption of the biocatalysts on the g‐C_3_N_4_ surface. The cascade reaction using SAS, however, resulted in a higher concentration of the desired enantiomerically pure amines. Nonetheless, the obtained product formations obtained with SAS were lower than expected. It could be shown that in the one‐pot one‐step procedure, some limitations were faced: 1) an oxidative inactivation and degradation of the biocatalyst by photoexcited SAS and the used light occurred; 2) the rate of the SAS‐catalyzed oxidation of the substrate was much slower in the one‐step one‐pot approach, most probably due to SAS's activity on the amine donor and the product of the reductive amination step. Thus, a one‐pot two‐step procedure was established, wherein first, the photochemical reaction was performed, followed by the subsequent addition of the biocatalyst for the reductive amination in a second reaction step. With this approach, around 4.5 mM of the desired amine with an *ee* of >99 % could be achieved. To further explore the scope of this sequential cascade, aliphatic, aromatic, chiral and nonchiral substrates were investigated. After the photooxidation step was performed, the reductive amination step yielded in 8.2 mM (with *AT*ωTA) and 11.97 mM (with *BM*ωTA), respectively, of amine when 3‐chlorobenzaldehyde was the substrate. Despite the good yields (>80 %) achieved with some of the aromatic substrates, however, a decreased yield was observed when increasing the length of the aliphatic side chain. Even though operating the PCE reaction is in principle feasible, such a combination remains challenging due to the inactivation effects of the biocatalyst by the photoexcited SAS. A separation of the photocatalyst and the biocatalyst in the reaction mixture, for instance, by immobilized catalysts or a flow chemistry setup, might overcome this problem.

**Scheme 13:**
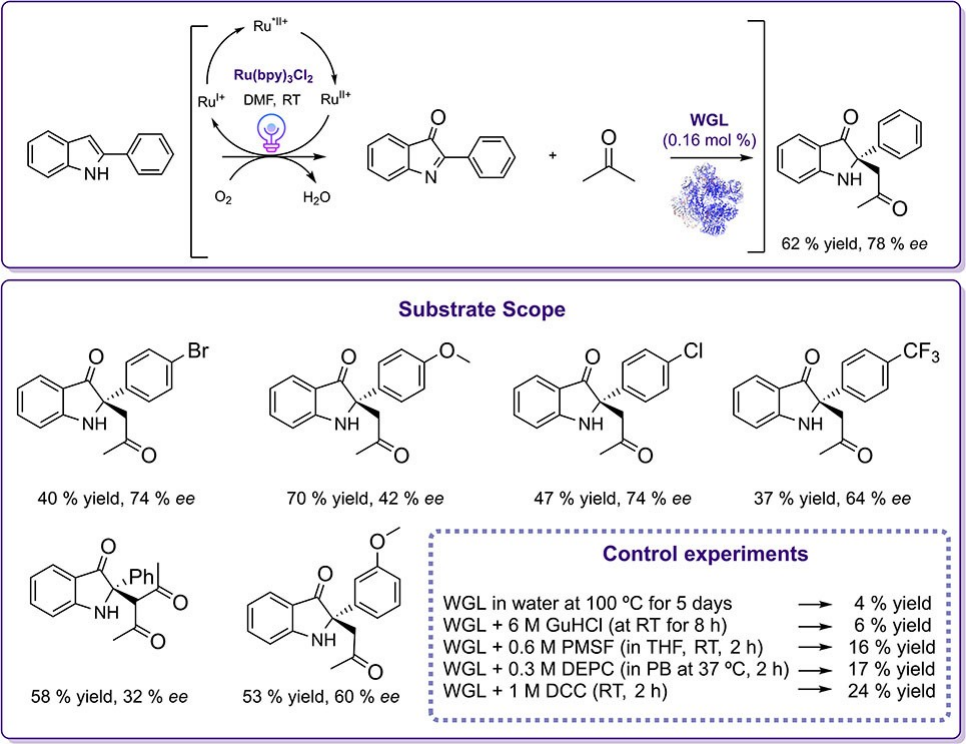
Photo‐biocatalytic synthesis of 2,2‐disubstituted indol‐3‐ones from 2‐arylindoles. Reaction conditions: substrate (0.3 mmol), acetone (24 mmol), enzyme (70 mg, 0.16 mol %), and Ru(bpy)_3_Cl_2_ ⋅ 6H_2_O (2 mol %) in DMF irradiated with a 32 W compact fluorescent lamp (CFL) at RT for 70 h.^[112]^

For the synthesis of asymmetric 2,2‐disubstituted indol‐3‐ones from 2‐arylindoles, a concurrent cascade was designed by coupling a photocatalytic oxyfunctionalization with the subsequent enantioselective alkylation catalyzed by hydrolases (Scheme 13).^[112]^ For a first proof of concept, 2‐phenylindole and acetone were chosen as substrates. Previously, it has been reported that 2‐arylindoles can be oxidized to indol‐3‐ones by visible light photoredox catalysis.^[113]^ Thus, the authors investigated the photooxidation step with Ru(bpy)_3_Cl_2_ as photocatalyst to generate the intermediate indolone, which serves as substrate for the lipase‐catalyzed asymmetric alkylation step.

Under non‐optimized conditions, this cascade reaction resulted in a yield of 12 % of the desired product with an enantiomeric ratio of 90 : 10. In order to optimize the system, a solvent screening was performed, and the effects of enzyme loading, photocatalyst loading, amount of acetone, DMF volume, light source, and reaction time on the photoenzymatic reaction were investigated. Under optimized conditions, the model reaction afforded the desired product with 62 % yield with 89 : 11 enantiomeric ratio. Extension of the substrate scope to the reactivity of different 2‐arylindoles and ketones was also possible. Additionally, the effect of different inhibitors on the catalytic activity of WGL was investigated. Several control experiments with WGL using the inhibitors were carried out. It could be revealed that the residues of the catalytic triad are vital for the catalytic activity of WGL in this photoenzymatic concurrent reaction (Scheme 13).

Nowadays, oils and fats are considered as an important source of renewable raw materials. Biocatalytic approaches can facilitate the first‐stage valorization in order to use these renewable resources for the production of fine chemicals.

**Scheme 14:**
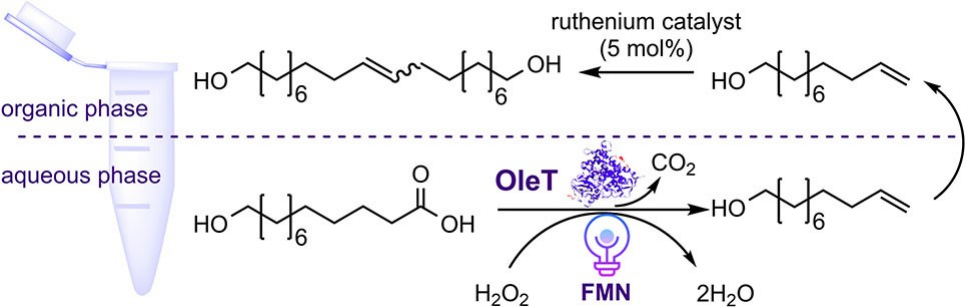
Schematic representation of the sequential photo‐chemoenzymatic cascade reaction consisting of a light‐driven decarboxylation step catalyzed by OleT that was coupled to a ruthenium catalyst for the synthesis of long‐chain alkendiol from bio‐based fatty acids. The first reaction step proceeded in the presence of isooctane to assist the extraction of intermediate ω‐alkenol into the organic phase.^[114]^

Bojarra and co‐workers designed a PCE cascade for the synthesis of ω‐unsaturated fatty acids, alkenylamines and long‐chain alkendiols from ω‐functionalized fatty acids by employing the fatty acid decarboxylase OleT from *Jeotgalicoccus* sp. ATCC 8456.^[114]^ The decarboxylation of ω‐hydroxy fatty acids of various chain lengths leading to the corresponding ω‐alkenols was accomplished with an in situ light‐driven H_2_O_2_ generation system using FMN and EDTA as sacrificial electron donor. Despite the production of ω‐alkenols, the formation of side‐products, which was an unexpected hurdle, significantly lowered the overall amount of obtained ω‐alkenols. Thus, reaction conditions were optimized to achieve higher formations of the corresponding products. With the optimized light‐driven decarboxylation system, 2 mM ω‐hydroxyhexadecanoic acid was successfully converted to the ω‐alkenol within 20 h. To further functionalize the obtained ω‐alkenols, this photo‐biocatalytic OleT reaction was coupled to either an ADH or alcohol oxidase as well as an ATA for the subsequent oxidation of the terminal alcohol and a subsequent reductive transamination. The reactions could be performed either sequentially or simultaneously. However, running the cascade in a two‐pot two‐step fashion with intermediary extraction resulted in better results than the one‐pot reaction. The authors contributed this to substantial oxidation to undec‐10‐en‐1‐oic acid, which reduced the amine formation in the one‐pot reaction. Finally, the authors investigated a Ru‐catalyzed olefin metathesis to synthesize long‐chain terminal diols from the decarboxylation products (Scheme 14). As the biphasic system consisting of tris buffer and isooctane exhibited high efficiency, three different cascade approaches were conducted either simultaneously or sequentially:1) sequential two‐pot mode, 2) sequential one‐pot mode, 3) simultaneous one‐pot mode. The sequential two‐pot mode, in which the decarboxylation catalyzed by OleT was performed in purely aqueous buffer followed by a subsequent extraction step, was considered as economically unfavorable. In the second reaction, the enzymatic decarboxylation was performed in the presence of isooctane as the second phase or in purely aqueous buffer with the addition of the ruthenium catalyst, allowing an enzymatic conversion of up to 90 %. In the final mode, a simultaneous one‐pot metathesis reaction encompassing a biphasic buffer/isooctane system was performed, and the upper organic phase was protected from light to prevent possible light degradation. However, the conversion remained lower than 20 %, which was attributed to the incompatibility between the cell lysate and the metathesis catalyst. Based on the obtained results, the sequential cascade approach with the extraction of the intermediate into the organic phase, followed by a suitable metathesis reaction, is obviously the most convenient cascade mode (Scheme 14).

### 3.2 Photoenzymes in biocatalytic cascades

Hybrid enzymes that were developed for the direct photoactivation of the biocatalyst (as described in Section 2.6) were also applied in PCE cascade reactions for the synthesis of trifluoro methylated/hydroxylated substituted arenes.^[115]^ The proof‐of‐concept was performed with a hybrid P450 BM3 catalyst containing a covalently attached Ru^II^‐diimine photosensitizer. The Ru^II^‐diimine photosensitizer initiates single electron transfer events and under photoredox conditions, a CF_3_ radical can add to arenes. The covalently attached Ru^II^‐diimine photosensitizer in the P450 BM3 hybrid enzyme provides the necessary electrons to perform, upon visible light activation, hydroxylation reactions on the trifluoromethylated substrates. A range of different substrates was explored and despite of obtaining low yields with some of the investigated substrates, the regio‐ and stereoselectivity of the hybrid P450 catalyst that differentiates between the trifluoromethylated isomers represents a benefit in the system. However, the need to purify the mixture of trifluoromethylated products to obtain only the desired isomers is a drawback and may be overcome by further protein engineering of the hybrid catalysts.

**Scheme 15:**
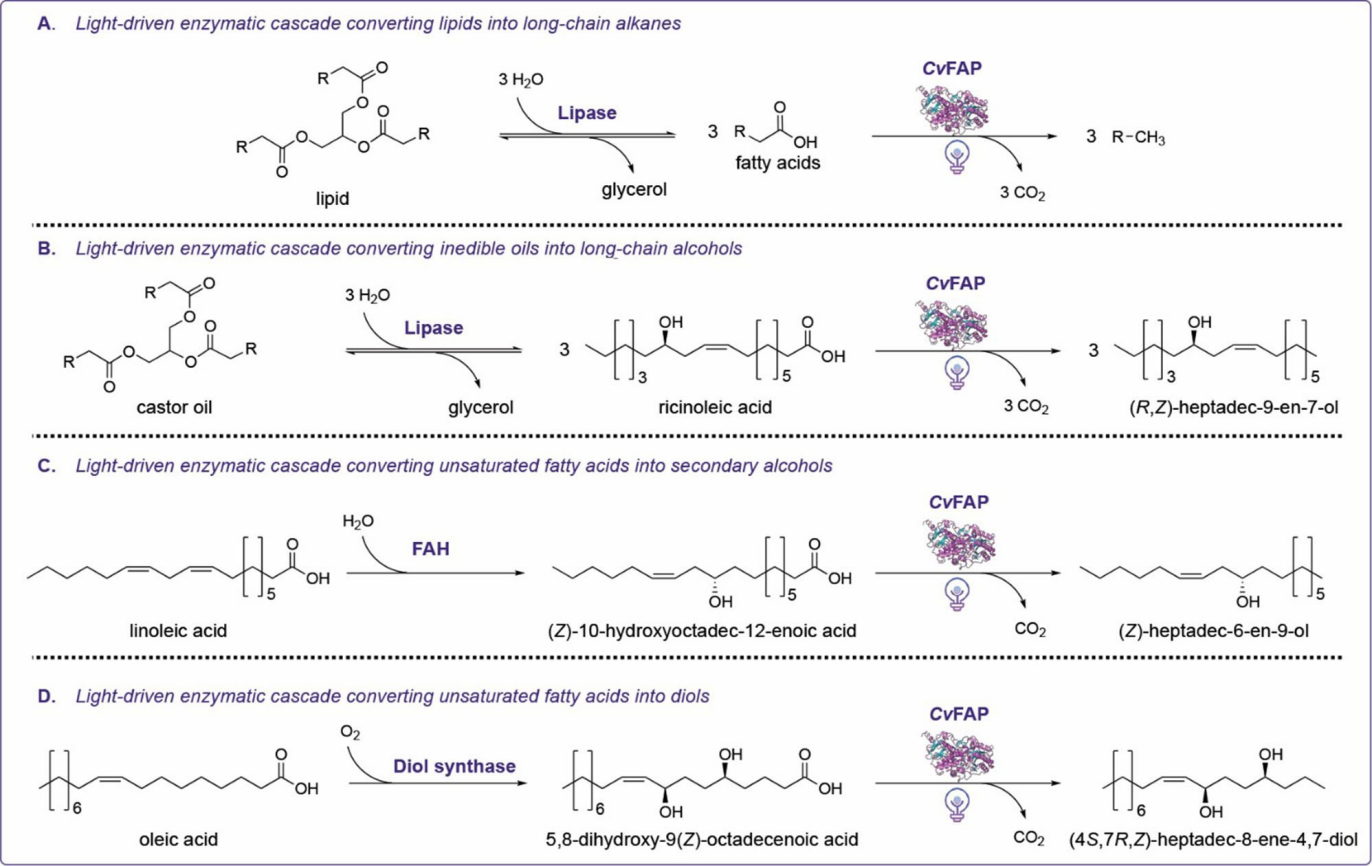
Different photo‐biocatalytic cascades comprising a photo‐decarboxylation step catalyzed by *Cv*FAP.[^92^, ^93^, ^116^]

The use of “true” photoenzymes capable of the immediate conversion of light into chemical energy is of considerable interest aiming at simplified photo‐biocatalytic reactions schemes. Huijbers and co‐workers showed for the first time the application of *Cv*FAP from *Chlorella variabilis* in a cascade reaction.^[93]^ The feasibility of converting saturated as well as unsaturated fatty acids into the corresponding long‐chain alkanes or alkenes by a light‐driven decarboxylation catalyzed by *Cv*FAP was first confirmed.^[93]^ As an alternative to the typical transesterification that is applied in biofuel production, the generation of long‐chain alkenes from triglycerides by an enzymatic two‐step cascade combining the enzymatic hydrolysis of triglycerids with subsequent light‐driven decarboxylation catalyzed by *Cv*FAP was shown (Scheme 15A).[^92^, ^93^] As reported, a homogeneous reaction system comprising the lipase *Cr*LIP from *Candida rugosa* and *E. coli* cell extract containing *Cv*FAP that was irradiated with blue LED light yielded an overall conversion of up to 83 % and an already promising turnover number of 8280 at a substrate concentration of 20 mM triolein.^[93]^ In a follow‐up study, the feasibility of producing alkanes originated from different natural (waste) oils was evaluated by performing whole‐cell biotransformations under blue‐light exposure. As indicated by the authors, product concentrations of up to 24 g L^−1^ could be achieved within 48 h.^[92]^ Besides the described production of alkanes from triglycerides, Ma and co‐workers proposed a bienzymatic cascade converting castor oil into (*R*,*Z*)‐octadec‐9‐en‐7‐ol (Scheme 15B).^[95]^ Thereby, initial hydrolysis of inedible castor oil by a lipase yields in the formation of free ricinoleic acid which then serves as a substrate for the decarboxylation catalyzed by *Cv*FAP under blue‐light irradiation yielding in product concentrations of up to 60 mM.^[95]^ As emphasized by the authors, a further optimization of the respective enzyme cascade is crucial to compensate for the drop in the pH value due to the accumulation of fatty acids in the reaction mixture and thus would improve the overall product yield.[^93^, ^95^] Zhang and co‐workers recently proposed the light‐driven biotransformation of unsaturated fatty acids into secondary fatty alcohols in a one‐pot two‐step enzymatic cascade.^[116]^ In a first step, hydration of unsaturated long‐chain fatty acids catalyzed by fatty acid hydratases (FAHs) yields in the formation of hydroxylated carboxylic acid intermediates serving as substrates for the following photoenzymatic decarboxylation step catalyzed by *Cv*FAP. Thereby, product conversions of up to 90 % could be achieved, providing 5 mM of unsaturated fatty acids as product. In a semipreparative light‐driven biotransformation with polyunsaturated linoleic acid as a substrate, 82.5 mg corresponding to an isolated yield of 32.5 % of optically pure secondary alcohol could be obtained (Scheme 15C).^[116]^ In the same study, the di‐hydroxylation of oleic acid was shown by using a three‐step reaction comprising a 5,8‐diol synthase and *Cv*FAP that yielded in the formation of up to about 10 mM (*Z*)‐heptadec‐8‐ene‐4,7‐diol product (Scheme 15D).^[116]^

### 3.3 Photo‐chemoenzymatic cascades used in PEC platforms, as biosensors and in bioassays

In contrast to sequential or simultaneous PCE cascades developed for the production of high‐value‐added compounds as discussed above, PCE approaches have also been developed together with PECs or were applied as biosensors and for bioassays. In the following, we will shortly outline a few examples that combine photo(electro)chemical with enzymatic transformations as another field of application.

The first example, comprising a PEC and an integrated enzyme cascade was developed for the reduction of CO_2_ to methanol.^[117]^ NADH was recycled using photogenerated electrons derived from the oxidation of water, fueled by solar‐light energy in order to transfer the electrons to the multienzymatic cascade consisting of three different enzymes, namely formate dehydrogenase (FDH), formaldehyde dehydrogenase (FaldDH) and ADH for the reduction of CO_2_. After optimization of reaction conditions, a Co−Pi/α‐Fe_2_O_3_ photoanode extracted the electrons from water oxidation, and the photoexcited electrons were further transferred to the photocathode (BiFeO_3_) within the two compartments of the PEC cell. Subsequently, a rhodium‐based mediator, [CpRh(bpy)(H_2_O)]^2+^, reduced the NAD^+^ to NADH. Ultimately, the excited electrons from reduced NAD^+^ were delivered to the three‐enzyme cascade containing FDH/FaldDH/ADH for the biocatalytic synthesis of methanol from the conversion of formate in a step‐wise manner, thereby leading to the average rate of methanol formation of 220 μM h^−1^.

Similar to this work, a bi‐enzyme PEC cascade‐based biosensor was designed for the detection of glucose.^[118]^ An initial photocurrent signal was modulated by g‐C_3_N_4_/ZnIn_2_S_4_ composite followed by a subsequent reaction for the biocatalytic oxidation of glucose by glucose oxidase leading to the formation of H_2_O_2_, which then oxidizes 4‐chloro‐1‐naphthol to generate a precipitate in the presence of horseradish peroxidase.

Another example describes photoactive nano‐complexes consisting of PbS QD‐sensitized inverse opal TiO_2_ electrodes (IO‐TiO_2_) coupled to FAD‐dependent glucose dehydrogenase (FAD‐GDH), which provides an efficient electron transfer platform for the light‐driven oxidation of glucose.^[119]^ The biohybrid signal chain activated by light enabled an electron transfer mechanism from the enzyme towards the redox polymer via the QDs, and subsequently to the IO‐TiO_2_ electrode, showing a concentration‐dependent behavior between 10 μM and 50 mM glucose. Particularly, the signal response of photocurrent can be regulated by the light intensity, the wavelength used during irradiation and the glucose concentration, thus facilitating the control of the biocatalytic reaction at the electrode interfaces. The last example is a photoswitchable enzyme cascade that was developed for signal amplification in a tyrosinase‐based colorimetric bioassay.^[120]^ Therein, the tyrosinase catalyzes the generation of dihydroxyphenylalanine (DOPA) coordinated TiO_2_ nanoparticles to form a light responsive nano‐trigger, which subsequently photoactivates a horseradish peroxidase (HRP). As the nano‐trigger (TiO_2_/DOPA) is in situ formed by the tyrosinase‐catalyzed reaction, which enabled the coupling of the tyrosinase/HRP reaction, a signal amplification was achieved.

## 4 Current Challenges of Applying Light in Biocatalysis

The variety of the hitherto developed photo(organo)catalytic reactions which use light to directly drive small molecule interconversions with further enzymatic functionalization steps in photo‐biocatalytic cascades impressively shows the powerfulness of this approach. However, it should be noted that, apart from a few exemptions, most of the cascades were performed with substrate concentrations in the lower‐millimolar range, and a (semi)preparative scale reaction could be demonstrated only in a few cases. The reasons for this are most probably associated with the current limitations, which we shortly outline in the following, on the way to developing efficient PCE cascade reactions.

Although many of the herein described PCE cascade reactions have been conducted in a simultaneous, that is, in a one‐pot one‐step fashion, only a handful of these examples operate without any loss of efficiency in this mode. One of the main reasons for a loss of efficiency in a simultaneous operation mode is the insufficient compatibility of the reaction conditions that are necessary for the photocatalyst and the enzyme, which represents a common problem when performing chemoenzymatic approaches. Thus, it is not surprising that the performance of PCE cascades was often increased when the reaction is performed in a two‐phase system or when the photocatalytic and the biocatalytic reaction are separated either spatially or temporally. As the prediction of compatibility issues between the photocatalyst and the enzyme can be challenging, many cascade reactions need to be elaborately investigated under different conditions to find the most suitable operation parameters. In order to overcome these limitations, a range of different solutions has been suggested, that includes protein engineering to increase the resistance of the biocatalyst, flow chemistry setups, catalyst immobilization or compartmentalization approaches. Whereas protein engineering might be tedious in order to make an enzyme more resistant against the sometimes harsh reaction conditions that a photocatalyst requires, flow chemistry approaches have already been successfully applied in photocatalytic systems.[^121^, ^122^] Thus, the development of continuous‐flow conditions for PEC cascades might be a promising solution to overcome compatibility issues. Also, compartmentalization approaches using whole cells have been investigated.[^45^, ^77^, ^106^] Using the protective environment of whole cells containing the biocatalyst, the enzyme stability is increased^[45,77,78],^ or the cell impedes that a photocatalyst diffuses through the cell membrane and thus ensures the spatial separation of the photo‐ and biocatalytic reaction.[^106^, ^123^] By that, other limitations as the intracellular regeneration of expensive cofactors could be circumvented.^[124]^ In addition to the mostly different conditions of the reaction medium, the light component often causes unexpected side effects, such as the generation of reactive radical species by excited photosensitizers.^[31]^ However, not only the choice of photocatalyst or photosensitizer/mediator influences the outcome of a certain reaction, also the choice of the sacrificial electron donor and the photosensitizer is crucial and has a significant impact on the overall reaction and thus needs to be carefully evaluated.[^45^, ^125^] Most of the applied light‐driven reaction systems reported in the literature rely on the presence of alternative simple sacrificial electron donors such as EDTA, TEOA or TEA. These tertiary amines show sufficient quenching abilities to various excited photosensitizers such as flavins,[^48^, ^56^, ^68^] organic dyes,[^45^, ^77^] carbon dots^[70]^ or polypyridine complexes.[^53^, ^126^] However, the formation of unfavorable decomposition products and operational pH dependency have to be considered by the use of tertiary amines as sacrificial electron donors.[^127^, ^128^] Besides low atom economy,^[48]^ the capability of EDTA to sequester metal ions which potentially can affect the functionality of metal‐containing biocatalysts such as the heme‐containing CYPs.^[129]^ As observed for tertiary amines, the use of ascorbic acid as sacrificial electron donor yields in the generation of undesired degradation products. Thereby, oxidation of ascorbic acid generates dehydroascorbic acid, an oxidant that can interfere with other components such as reduced photosensitizer or substrate.^[128]^ Recent studies also propose the use of redox‐active buffer agents like MES, MOPS or HEPES as potential reductants in light‐driven biotransformations.^[35]^ These compounds show no toxic effects and can be taken up by *E. coli* cells enabling their applications in *in vitro*[^35^, ^130^] and *in vivo* studies^[45]^ at a constant pH value.

In order to avoid any problems caused by the use of sacrificial organic donors, the use of H_2_O is of great interest, also in regard to costs, atom efficiency, biocompatibility and sustainability. However, only a few proof of concept studies reported the feasibility of using H_2_O in combination with inorganic photocatalysts such as TiO_2_ to perform light‐driven biotransformations. The high stability and low oxidation potential often hamper a general applicability of water as a suitable electron donor for photo‐biocatalytic approaches.^[52]^

Up to now, a wide range of organic photosensitizers, QDs and carbon‐based nanomaterials have been successfully implemented in photo‐biocatalytic approaches. Depending on their application, each of these photosensitizers offers several benefits, but also disadvantages. Organic dyes, for instance, are prone to inactivation due to photobleaching.[^45^, ^131^, ^132^, ^133^] QDs are often based on toxic heavy metals like cadmium which is unbeneficial for their implementation in environmental benign processes and carbon‐based nanoparticles such as g‐C_3_N_4_ have been reported to bind to the biocatalyst or causes locally high concentrations of radicals which leads to the inactivation of the enzyme.^[134]^

A further challenge on the way to a wide applicability of photo‐chemo‐biocatalytic concepts has been recently emphasized by Edwards and co‐workers and concerns the equipment setup used for photocatalytic reactions.^[135]^ In order to ensure that photochemical approaches can be reproduced in every laboratory, a deeper characterization of the often home‐made light reactor setups must be performed to enable a greater mechanistic understanding of the underlying photochemical principles and thus to pave the way for scaling up photo‐biocatalytic approaches. Especially differences caused by the light source of choice can cause batch‐to‐batch variability. Thus, it is of great importance to gather knowledge on the light source used, photon stoichiometry, internal reaction temperature, light intensity, the distance between the light source and reaction mixture, and path length.^[135]^ As Edwards and co‐workers highlighted, a trend towards standardization of photochemistry platforms is there, which is crucial to ease a broader usage of photochemistry across academia and industry.^[135]^

## 5 Summary and Outlook

Biocatalysis has emerged as an environmentally friendly technology for the chemical and pharmaceutical industries. A large series of successful examples underline that biocatalysis can improve the sustainability of chemical processes and thus complements other catalytic technologies very successfully. The combination of redox biocatalysis with photocatalysis by harnessing the energy from photon absorption to drive chemical transformations represents a powerful strategy to contribute to an even “greener” chemistry. Thus, it is not surprising that manifold concepts for the utilization of light as a driving force for enzyme‐catalyzed reactions have been developed in the past years. Although the majority of examples focus on light‐driven cofactor‐regeneration strategies, these approaches suffer from low efficiency of the enzymes and thus result in low turnover numbers, which impedes their applicability. However, several recent examples with a focus on PCE cascade reactions, the discovery of natural or engineered photoenzymes as well as the exploitation of promiscuous enzyme activities in the presence of light might highlight the true power of photo‐biocatalysis. The future will unravel the full potential of these newly developed strategies for organic synthesis and together with the development of new and efficient photobioreactor concepts for up‐scaling, it is expected that the field can be pushed forward to the next level.

## Conflict of interest

The authors declare no conflict of interest.

## Biographical Information

*F. Feyza Özgen received her B.Sc. and M.Sc. degrees in genetics and bioengineering at Istanbul University in 2017. During the latter, she was a project assistant under the supervision of Assoc. Prof. Gönül Schara in the field of protein engineering. In 2018, she started her Ph.D. as an early‐stage researcher within the MSCA‐ITN PhotoBioCat under the guidance of Assist. Prof. Sandy Schmidt at the Rijksuniversiteit Groningen. During her project, she completed an academic secondment at Graz University of Technology and studied at Aix‐Marseille Université. Her research interests include enzyme engineering, the development of photo‐biocatalytic cascade reactions and light‐driven Rieske‐type hydroxylations*.

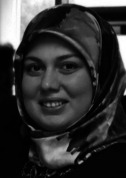

## Biographical Information

*Michael E. Runda received a B.Sc. degree in molecular biology at the University of Graz in 2016, followed by a M.Sc. in biotechnology at Graz University of Technology in 2019 on the photoactivation of Rieske enzymes by the implementation of a novel light‐driven electron pathway within non‐autotrophic organisms. In 2020, he started his Ph.D. program at the Department of Chemical and Pharmaceutical Biology at the Groningen Research Institute of Pharmacy. Under the supervision of Prof. Gerrit J. Poelarends and Assist. Prof. Sandy Schmidt, he is currently elucidating the contribution of oxygenases in the late‐stage diversification of natural products*.

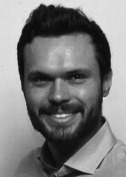

## Biographical Information

*Sandy Schmidt completed her Ph.D. in 2015 in the group of Prof. Uwe Bornscheuer at the University of Greifswald in the field of protein engineering and enzymatic cascade reactions. After a research stay at Delft University of Technology as postdoctoral fellow within the group of Prof. Dr. Frank Hollmann, she worked as a group leader at Graz University of Technology. Since April 2020, she has been Assistant Professor and Rosalind Franklin Fellow at the Rijksuniversiteit Groningen. Her research interests focus on photo‐biocatalysis, synthetic metabolic pathways and multienzymatic cascades. Moreover, she investigates structure‐function relationships of dioxygenases as well as electron transfer pathways in oxidoreductases and light‐driven biocatalytic processes*.